# Forecasting Fetal Buprenorphine Exposure through Maternal–Fetal Physiologically Based Pharmacokinetic Modeling

**DOI:** 10.3390/pharmaceutics16030375

**Published:** 2024-03-08

**Authors:** Matthijs W. van Hoogdalem, Ryota Tanaka, Khaled Abduljalil, Trevor N. Johnson, Scott L. Wexelblatt, Henry T. Akinbi, Alexander A. Vinks, Tomoyuki Mizuno

**Affiliations:** 1 Division of Translational and Clinical Pharmacology, Cincinnati Children’s Hospital Medical Center, Cincinnati, OH 45229, USA; mvanhoo1@its.jnj.com (M.W.v.H.); rtanaka@oita-u.ac.jp (R.T.); sander.vinks@cchmc.org (A.A.V.); 2 James L. Winkle College of Pharmacy, University of Cincinnati, Cincinnati, OH 45229, USA; 3 Certara UK Limited, Simcyp Division, Sheffield S1 2BJ, UK; khaled.abduljalil@certara.com (K.A.); trevor.johnson@certara.com (T.N.J.); 4 Division of Neonatology, Perinatal Institute, Cincinnati Children’s Hospital Medical Center, Cincinnati, OH 45229, USA; scott.wexelblatt@cchmc.org (S.L.W.); henry.akinbi@cchmc.org (H.T.A.); 5 Department of Pediatrics, College of Medicine, University of Cincinnati, Cincinnati, OH 45267, USA; 6 Center for Addiction Research, College of Medicine, University of Cincinnati, Cincinnati, OH 45267, USA

**Keywords:** buprenorphine, maternal–fetal, physiologically based pharmacokinetic modeling, neonatal opioid withdrawal syndrome, opioid use disorder

## Abstract

Buprenorphine readily crosses the placenta, and with greater prenatal exposure, neonatal opioid withdrawal syndrome (NOWS) likely grows more severe. Current dosing strategies can be further improved by tailoring doses to expected NOWS severity. To allow the conceptualization of fetal buprenorphine exposure, a maternal–fetal physiologically based pharmacokinetic (PBPK) model for sublingual buprenorphine was developed using Simcyp (v21.0). Buprenorphine transplacental passage was predicted from its physicochemical properties. The maternal–fetal PBPK model integrated reduced transmucosal absorption driven by lower salivary pH and induced metabolism observed during pregnancy. Maternal pharmacokinetics was adequately predicted in the second trimester, third trimester, and postpartum period, with the simulated area under the curve from 0 to 12 h, apparent clearance, and peak concentration falling within the 1.25-fold prediction error range. Following post hoc adjustment of the likely degree of individual maternal sublingual absorption, umbilical cord blood concentrations at delivery (n = 21) were adequately predicted, with a geometric mean ratio between predicted and observed fetal concentrations of 1.15 and with 95.2% falling within the 2-fold prediction error range. The maternal–fetal PBPK model developed in this study can be used to forecast fetal buprenorphine exposure and would be valuable to investigate its correlation to NOWS severity.

## 1. Introduction

The transfer of xenobiotics across the human placenta is well described [1]. Although the placenta acts as a barrier for some compounds, virtually all drugs ultimately reach the fetal circulation to a certain extent. Transplacental exchange occurs predominantly via passive diffusion, but other mechanisms, such as active transport, facilitated diffusion, and, for specific endogenous compounds, phagocytosis and pinocytosis can be observed [2,3]. The safety of the developing fetus is of foremost concern with any drug use by the expectant mother. The effects of fetal exposure to xenobiotics can manifest in various forms. In severe cases, drugs are teratogenic, arguably most notoriously exemplified by thalidomide [4]. Other substances can cause neonatal withdrawal following childbirth, since the supply abruptly halts with the clamping of the umbilical cord. The incidence of a specific type of neonatal withdrawal, namely neonatal opioid withdrawal syndrome (NOWS), continues to rise as the opioid crisis spreads across the US [5].

The severity of NOWS differs significantly between neonates. The symptoms of some newborns can be controlled through nonpharmacological approaches, while additional pharmacotherapy is warranted for others [6]. The pharmacological treatment of NOWS primarily encompasses the administration of opioids to control withdrawal symptoms [7]. Buprenorphine has become the preferred choice for medication-assisted treatment for opioid use disorder in pregnant women [8]. Buprenorphine administered as a sublingual solution is an emerging treatment for NOWS [9]. However, dosing is currently empiric, as individualized evidence-based precision dosing strategies are still under development [10,11,12,13]. The tailoring of (starting) doses to the expected NOWS severity represents one method to refine treatment with buprenorphine. The accurate prediction of NOWS severity has the additional potential benefit of objectively predicting the course of an individual postnatal observation period in the nursery, which is currently arbitrarily set at 72–96 h to monitor for the development of withdrawal symptoms [14].

Various factors account for the variability in NOWS severity, but the principal driver seems to be the extent of in utero opioid exposure [15]. Sublingually administered buprenorphine is, besides its application in the treatment of NOWS, one of the most prescribed drugs to manage opioid use disorder in Medicaid enrollees [16] and part of the standard of care for opioid dependence during pregnancy. Predicting prenatal buprenorphine exposure is therefore of interest, as this could explain the variability in NOWS severity in neonates born to opioid-dependent mothers maintained on buprenorphine, which arguably forms a large portion of NOWS-diagnosed newborns.

Forecasting fetal cumulative buprenorphine exposure is challenging, since a kaleidoscopic interplay of maternal and fetal physiological factors, which constantly evolve throughout the gestational period, ultimately dictates concentration–time profiles in the fetus. Recently emerged maternal–fetal physiologically based pharmacokinetic (PBPK) modeling approaches offer a platform that integrates the characteristics, growth, and maturation of maternal and fetal physiology to predict fetal drug exposure [17,18]. The aim of the present study was to develop a maternal–fetal PBPK model for sublingual buprenorphine that can predict fetal exposure based on the maternal intake, building upon buprenorphine PBPK models previously developed by our group, namely a minimal neonatal PBPK model [12] and a full adult PBPK model [19]. The construction of such a model offers a framework to investigate the effect of fetal buprenorphine exposure on NOWS severity. Guiding NOWS treatment based on its expected severity has, in turn, the potential to improve clinical outcomes in NOWS-diagnosed newborns.

## 2. Materials and Methods

### 2.1. Maternal–Fetal PBPK Model Structure

**Figure 1:**
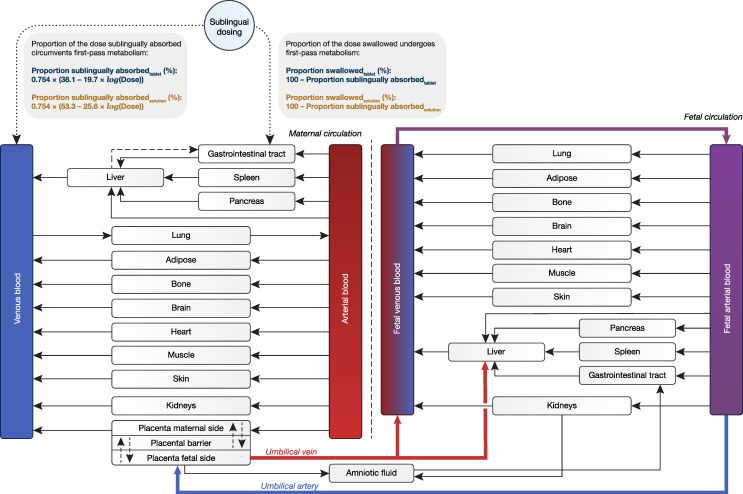
Structure of the full maternal–fetal physiologically based pharmacokinetic (PBPK) model. Since the sublingual route of administration is not available in Simcyp, sublingual absorption is mimicked by using the first-order inhalation model. In this model, the proportion of the dose inhaled equals the proportion sublingually absorbed, where the remaining fraction is swallowed.

A previously developed PBPK model for buprenorphine [19] in nonpregnant subjects was used as a base structural model. In the current work, this model was expanded to a maternal–fetal PBPK model within the Simcyp Simulator (v21.0; Simcyp Limited, Sheffield, UK). Drug physicochemical and physiological parameters used for maternal–fetal PBPK modeling are shown in Table S1. The maternal–fetal PBPK model structure is shown in Figure 1 and was obtained by employing the pregnancy module in Simcyp (Sim-Pregnancy population file) in combination with the permeability-limited placenta model [20]. Sublingual absorption was mimicked by using the first-order inhalation model in combination with the inhaled route of administration, as described previously [12,19]. A coefficient of variation (CV) of 33.9% was applied to the administered dose to reflect variability in bioavailability [19].

### 2.2. Effect of Salivary pH on Sublingual Absorption of Buprenorphine

Buprenorphine is an ampholyte that contains a tertiary amine and phenol group (p*K*_a_ = 8.31 and 9.62, respectively) [21] and therefore exists in a predominantly cationic state at physiological pH, where its net positive charge increases with decreasing pH. Salivary pH decreases during pregnancy [22], which may affect the sublingual absorption of buprenorphine, as reported by Mendelson et al., who investigated the relationship between individual salivary pH and recovery of buprenorphine in saliva (i.e., the inverse of sublingual absorption) [23]. Data points reported by Mendelson et al. were extracted using WebPlotDigitizer (v4.5, Ankit Rohatgi, Pacifica, CA, USA), and the correlation between salivary pH and the recovery of buprenorphine was explored through linear regression modeling using the stats package (v4.1.2, R Core Team) for R (v4.1.2, R Foundation for Statistical Computing, Vienna, Austria). The following bivariate linear model was used (Equation (1)):

Recovered (%) = α + β × pH(1)

where Recovered is the proportion of the sublingual dose recovered in saliva, α is the intercept, β is the slope, and pH is the salivary pH. Salivary recovery of buprenorphine was determined at pH 7.01 and 6.34, which are the salivary acidities in nonpregnant and pregnant women, respectively [22]. Previously developed nonlinear sublingual absorption models for buprenorphine [19], which describe the degree of sublingual absorption as a function of dose, were subsequently adjusted according to the difference in salivary recovery during pregnancy.

### 2.3. Buprenorphine Tissue Partitioning throughout Gestation 

In the nonpregnant adult PBPK model for buprenorphine developed previously, plasma-to-tissue partition coefficients (Kp) were estimated based on rat tissue distribution data [19]. However, the Rodgers and Rowland method (method 2) was used in this study to accommodate pregnancy-induced changes in maternal tissue partitioning (Figure S1) [24,25], with a Kp scalar of 0.26819 to recover the volume of distribution at steady state of the previously developed nonpregnant adult PBPK model (V_ss_ = 6.23 L/kg) [19]. Fetal Kp values were also calculated using method 2 and the aforementioned Kp scalar of 0.26819.

### 2.4. Pregnancy-Induced Changes in Enzyme Expression 

Besides standard pregnancy-associated enzyme induction profiles incorporated by default into the pregnancy module of Simcyp [26], increased expression of hepatic cytochrome P450 (CYP) 2C8 and intestinal CYP3A4 was assumed by copying the default induction profile of hepatic CYP3A4 to these enzymes. Likewise, hepatic UDP-glucuronosyltransferase (UGT) 2B7 was assumed to undergo the same pregnancy-associated induction trajectory as hepatic UGT1A1. For the fetus, standard enzyme induction profiles included in the Simcyp pregnancy module were used.

### 2.5. Placental Transfer of Buprenorphine 

To predict transplacental passive permeability (P_eff_) of buprenorphine, the following equation was used (Equation (2)): [27]

(2) $$P_{\mathrm{eff}}\;\left(10^{-4}\;\mathrm{cm}/s\right)=10^{1.454-0.011\;\times \;\mathrm{PSA}\;-0.278\times \;\mathrm{HDB}}$$

where PSA and HBD are buprenorphine’s polar surface area (PSA = 62.2 Å^2^) [28] and hydrogen bond donor count (HBD = 2) [28], respectively. Placental diffusion clearance (CL_PD_) was subsequently calculated as follows (Equation (3)): [18]

(3) $${\mathrm{CL}}_{\mathrm{PD}}\;\left(L/h/\mathrm{mL}\;\mathrm{placenta}\right)=P_{\mathrm{eff}}\times \frac{3.6\times \;\mathrm{PVSA}}{{\mathrm{Volume}}_{\mathrm{placenta}}}$$

where PVSA is the placenta villous surface area (in m^2^), Volume_placenta_ is the placental volume (in mL), and 3.6 is a unit conversion scalar (thus, unit of P_eff_ needs no conversion). PVSA and Volume_placenta_ were calculated following the default polynomials in Simcyp (Equations (4) and (5), respectively):

(4) $$\mathrm{PVSA}\;\left(m^{2}\right)=0.135\times \mathrm{GA}-0.023\times {\mathrm{GA}}^{2}+0.0015\times {\mathrm{GA}}^{3}-0.0002\times {\mathrm{GA}}^{4}$$

(5) $${\mathrm{Volume}}_{\mathrm{placenta}}\;\left(\mathrm{mL}\right)=1.0\times \mathrm{GA}+0.51\times {\mathrm{GA}}^{2}-0.0028\times {\mathrm{GA}}^{3}$$

where GA is the gestational age (in weeks). Thus, predicted CL_PD_ evolves throughout pregnancy due to PVSA and Volume_placenta_ changing with gestational age.

### 2.6. Verifying the Prediction of Maternal Pharmacokinetics (PK) during Pregnancy

Independent data (i.e., data not used in model development) reported by Zhang et al. [29], which emanated from a clinical trial conducted by Bastian et al. [30], were used for verification. Zhang et al. reported concentration–time profiles and area under the curve from 0 to 12 h (AUC_0–12h_), apparent clearance (CL/F), peak concentration (C_max_), and time to reach C_max_ (T_max_) for subjects in the second trimester, third trimester, and postpartum period [29]. Subjects received a stable twice-daily 8 mg buprenorphine dose as sublingual tablets. Ratios between predicted and observed (P/O ratios) AUC_0–12h_, CL/F, C_max_, and T_max_ were determined to verify the PBPK model’s predictive performance during pregnancy. WebPlotDigitizer was used to extract concentration–time data reported by Zhang et al. [29].

Virtual trials (10 mother–fetus dyads × 10 trials) were run to predict concentration–time profiles and PK parameters (defined as the geometric mean) of the virtual population. Virtual mother–fetus dyads were matched for maternal age, gestational age, and sublingual dose to those in the original trial. The duration of the virtual trial was set to 7.5 days, since participants in the original study received buprenorphine for at least 7 days, followed by a 12 h period to collect blood samples [29,30]. Time-varying covariates were accounted for in the model by using continuously updating physiological parameter values throughout the virtual trial to reflect growth and maturation during the study period. For simulation during the postpartum period, virtual women were created using the Sim-Healthy Volunteers population file, and no changes in salivary pH-dependent sublingual absorption and enzyme abundance were applied (i.e., the previously developed nonpregnant model was used) [19].

### 2.7. Verifying the Prediction of Fetal PK during Pregnancy

Bartu et al. [31] and Wiegand et al. [32] reported buprenorphine concentrations in maternal and umbilical cord blood at delivery in combination with maternal dosing data, and these independent data were used for verification. Pregnant women received between 1 and 28 mg buprenorphine daily as a sublingual tablet. The model’s predicted performance of fetal PK was assessed by comparing predicted and observed umbilical cord blood concentration–time points.

For all mother–fetus dyads of the original studies, 100 virtual twin mother–fetus dyads were created by matching the virtual cohort’s maternal age, gestational age, and sublingual dose to that of the original dyad. As described for maternal PK verification, current physiological values for the virtual fetuses were used to account for the developmental physiology over time, but here, virtual trials were run for 14 days to allow attainment of maternal and fetal pseudo-steady state, followed by at least 24 h to compare predicted vs. observed concentrations. For each virtual dyad, two simulations were run. One simulation was run in which maternal sublingual buprenorphine absorption was determined using the nonlinear absorption models corrected for more acidic salivary pH during pregnancy. To improve the model’s predicted performance for fetal concentration, another simulation was run in which the proportion sublingually absorbed by the mother was corrected post hoc across a range of 0.1–99.9% with the observed maternal concentration–time data. Finally, the maternal–fetal PBPK model was used to simulate maternal (aged 18–45 years) and umbilical cord blood concentrations at 15, 27, and 40 weeks of gestation following a standard 16 mg buprenorphine dose as a sublingual tablet. 

### 2.8. Statistical Analysis and Evaluation of Potential Bias

Geometric means and 95% confidence intervals (CIs) of concentration fold-differences were calculated using the DescTools package (v0.99.44, Signorell et mult. al.) for R. Normal distribution was examined through the Shapiro–Wilk test. The maternal–fetal PBPK model’s predictive performance during pregnancy was deemed adequate if predicted PK parameter P/O ratios fell between 0.8-fold and 1.25-fold of the observed value (1.25-fold prediction error range). The predictive performance of fetal PK was deemed adequate if, following post hoc optimization of maternal sublingual absorption, geometric mean umbilical cord blood concentrations fell within the 1.25-fold prediction error range. Additionally, the proportion of fetal concentration fold-differences falling within the wider 2-fold prediction error range was determined.

Potential bias in the model prediction was evaluated using goodness-of-fit plots for predicted vs. observed maternal and fetal concentrations.

## 3. Results

### 3.1. Reduced Sublingual Absorption of Buprenorphine Due to Lower Salivary pH during Pregnancy

Salivary recoveries of buprenorphine at pH 7.01 and 6.34 (i.e., salivary pH in nonpregnant and pregnant women, respectively) [22] were modeled at 54.5% and 65.7% (thus, proportions of dose sublingually absorbed were estimated at 45.5% and 34.3%), respectively (Figure S2), suggesting that pregnant subjects sublingually absorb 24.6% less than nonpregnant individuals. Hence, previously developed nonlinear absorption models for buprenorphine [19] were multiplied by 0.754 to describe the lower transmucosal absorption as a function of dose in pregnant women (Figure 1).

### 3.2. Maternal PBPK Model Prediction during Pregnancy

**Figure 2:**
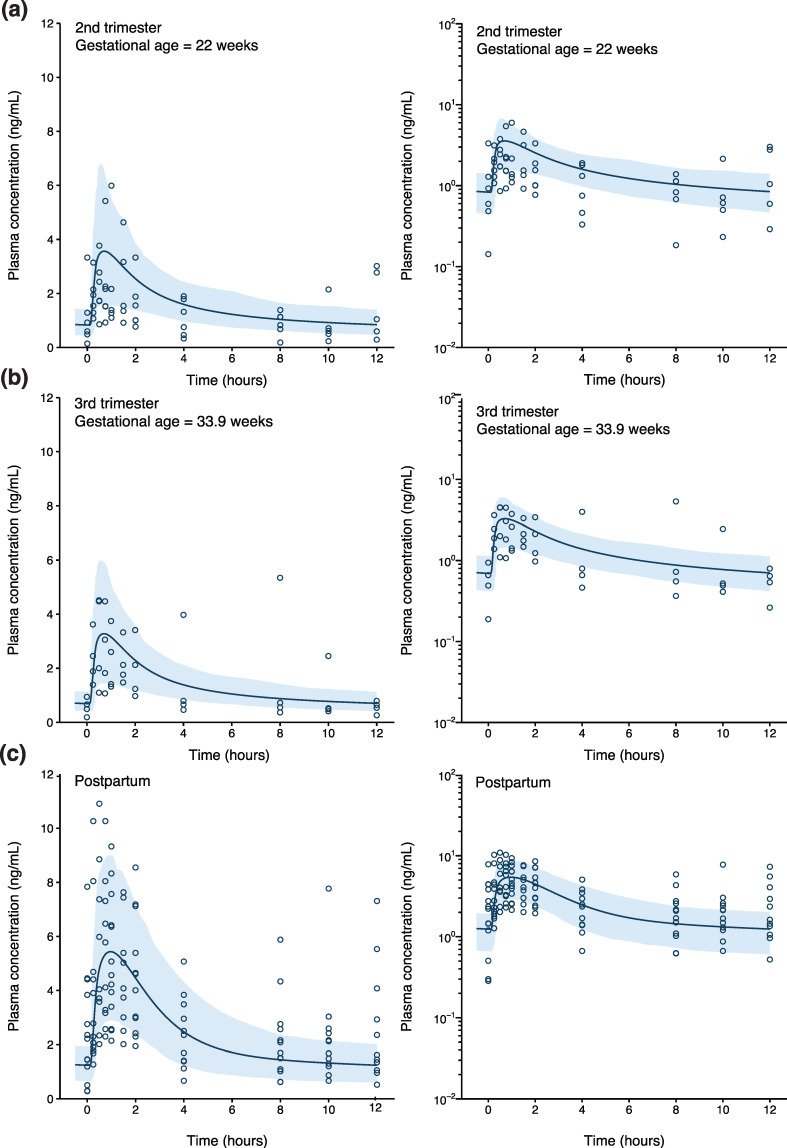
Maternal–fetal physiologically based pharmacokinetic (PBPK) model-based predicted and observed maternal buprenorphine plasma concentrations during the (**a**) second trimester, (**b**) third trimester, and (**c**) postpartum period. Pregnant subjects received 8 mg buprenorphine twice daily as sublingual tablets. Blue solid line and shaded area represent the mean concentration–time profile and 5th to 95th percentile range of the virtual population (n = 100), respectively. Open blue circles represent concentration–time data reported by Zhang et al. [29,30].

Following the incorporation of salivary pH-dependent reduced sublingual absorption and induced enzyme expression during pregnancy, model-predicted maternal concentration–time profiles and PK parameters for women in the second trimester (n = 4; mean gestational age = 22.0 weeks), third trimester (n = 4; mean gestational age = 33.9), and postpartum period (n = 10; on average 7.4 weeks after childbirth) were compared with observed data reported by Zhang et al. [29]. Maternal concentration–time profiles were adequately predicted in all periods (Figure 2). P/O ratios for AUC_0–12h_, CL/F, C_max_, and T_max_ fell within the 1.25-fold error range (Table S2), except for T_max_ during the second and third trimesters (P/O ratio = 0.73 and 1.58). 

### 3.3. Maternal–Fetal PBPK Model Prediction at Delivery

**Figure 3:**
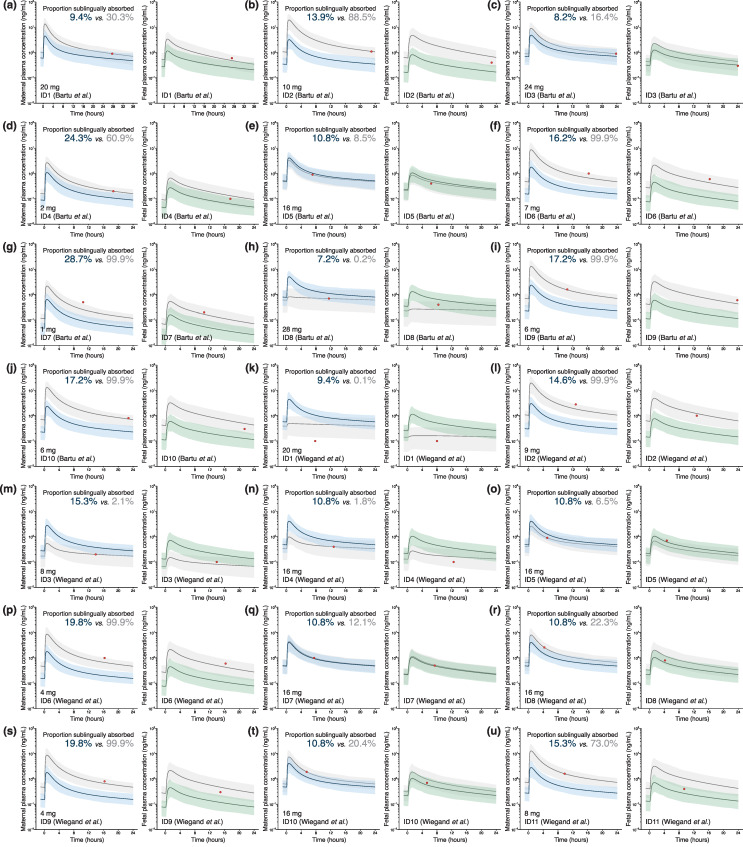
Individual maternal–fetal physiologically based pharmacokinetic (PBPK) model-based predicted and observed maternal and fetal buprenorphine plasma concentrations at delivery. Pregnant women received between 1 and 28 mg buprenorphine daily as sublingual tablets, and individual doses are shown in the lower left corner of each figure subsection. Closed red circles represent observed buprenorphine concentrations in maternal and umbilical cord blood reported by (**a**–**j**) Bartu et al. [31] and (**k**–**u**) Wiegand et al. [32] for a total of 21 mother–fetus dyads. Simulated concentration–time profiles with 5th to 95th population percentile ranges (n = 100 mother–fetus dyads) were created either under the presumption that the proportion of the administered dose sublingually absorbed by the expectant mother equals 0.754 × (38.1 − 19.7 × *log*(Dose)), which is the default absorption extent in the maternal–fetal PBPK model for sublingual tablets (shown in blue and green for maternal and fetal concentrations, respectively), or with the degree of maternal sublingual absorption optimized (across a range of 0.1–99.9%) post hoc to capture the reported maternal concentration–time point as accurately as possible (shown in grayscale). The final degrees of sublingual absorption are shown in the upper right corner of each figure subsection (written in blue and gray to reflect the model’s default and optimized value, respectively).

**Figure 4:**
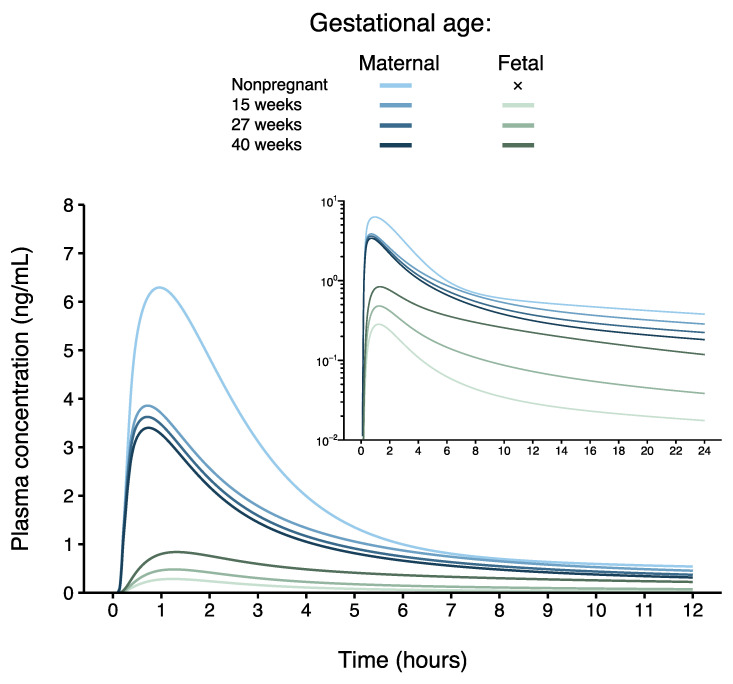
Mean maternal–fetal physiologically based pharmacokinetic (PBPK) model-based predicted maternal (aged 18–45 years) and umbilical cord blood concentrations of buprenorphine early in the second trimester, at the end of the second trimester, and at the end of the third trimester (15, 27, and 40 weeks’ gestational age, respectively) following a standard 16 mg buprenorphine dose as sublingual tablet. The concentration–time curves are juxtaposed with the expected profile of a nonpregnant woman under the same conditions (created using the previously developed base model) [19].

The PBPK-model-based predicted maternal and fetal concentration–time profiles were compared with individual observed profiles for a total of 21 mother–fetus dyads reported by Bartu et al. [31] and Wiegand et al. [32] (Figure 3). Following post hoc optimization of maternal sublingual absorption, umbilical cord blood concentrations were adequately predicted with a geometric mean (95% CI) fetal concentration fold-difference of 1.15 (0.98–1.35), and 20 out of 21 predicted fetal concentrations (95.2%) fell within the 2-fold prediction error range. Moreover, by grouping model-predicted and observed concentration profiles of mother–fetus dyads receiving identical buprenorphine dosages, followed by optimization of maternal sublingual absorption, the maternal–fetal PBPK model seemed to adequately capture the general shape of and variability in fetal concentration–time profiles (Figure S3). Simulation using the final maternal–fetal PBPK model indicated that maternal buprenorphine concentrations gradually decrease as pregnancy progresses, while fetal exposure conversely increases throughout gestation (Figure 4).

### 3.4. Evaluation of Bias in the Maternal–Fetal PBPK Model Prediction

**Figure 5:**
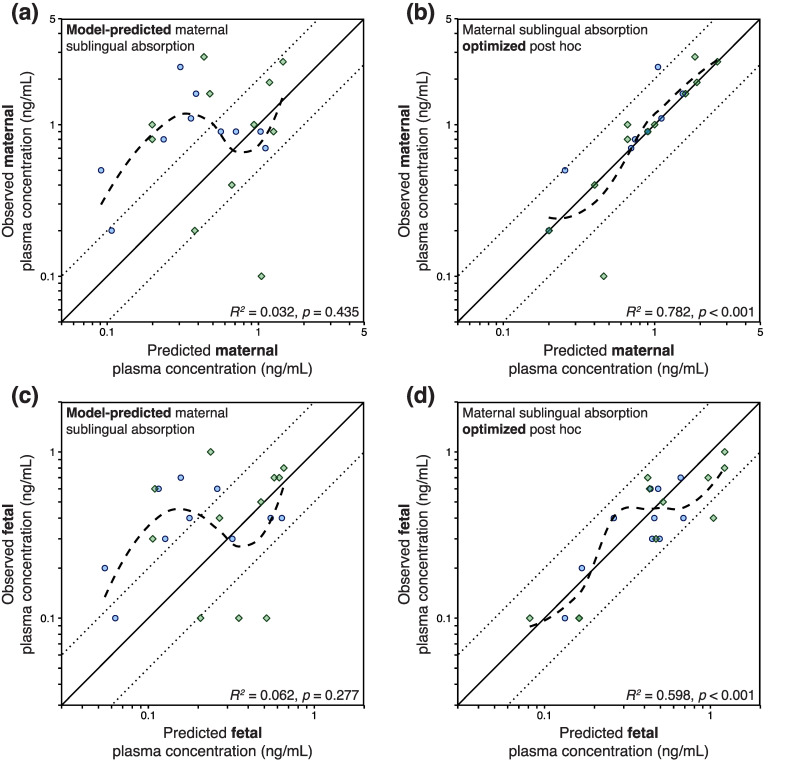
Goodness-of-fit plots for the maternal–fetal physiologically based pharmacokinetic (PBPK) model for buprenorphine, showing (**a**,**b**) predicted vs. observed maternal and (**c**,**d**) fetal concentrations following administration of buprenorphine sublingual tablets. Coefficients of determination (*R*^2^) and associated *p* values are shown in the lower right corner of each figure subset. Concentrations were predicted either (**a**,**c**) under the presumption that the proportion of the administered dose sublingually absorbed by the expectant mother equals 0.754 × (38.1 − 19.7 × *log*(Dose)), which is the default absorption extent in the maternal–fetal PBPK model for sublingual tablets, or (**b**,**d**) with the degree of maternal sublingual absorption optimized (across a range of 0.1–99.9%) post hoc to capture the reported maternal concentration–time point as accurately as possible. Blue circles (●) and green diamonds (◆) represent maternal and fetal concentration–time data reported by Bartu et al. [31] and Wiegand et al. [32], respectively. Dotted lines represent the 2-fold prediction error range. Curved dashed lines represent locally estimated scatterplot smoothing (LOESS) curves.

When employing the nonlinear absorption model for sublingual tablets [19] corrected for more acidic salivary pH during pregnancy, maternal plasma concentrations at delivery were generally underpredicted (Figure 5a). To improve this discrepancy, the proportion absorbed sublingually was individually modified by post hoc analysis with the observed maternal concentration. The post hoc optimization of maternal sublingual absorption across a range of 0.1–99.9% improved the prediction of maternal plasma concentrations (Figure 5b), which translated into refined prediction of umbilical cord blood concentrations (Figure 5c,d). 

**Figure 6:**
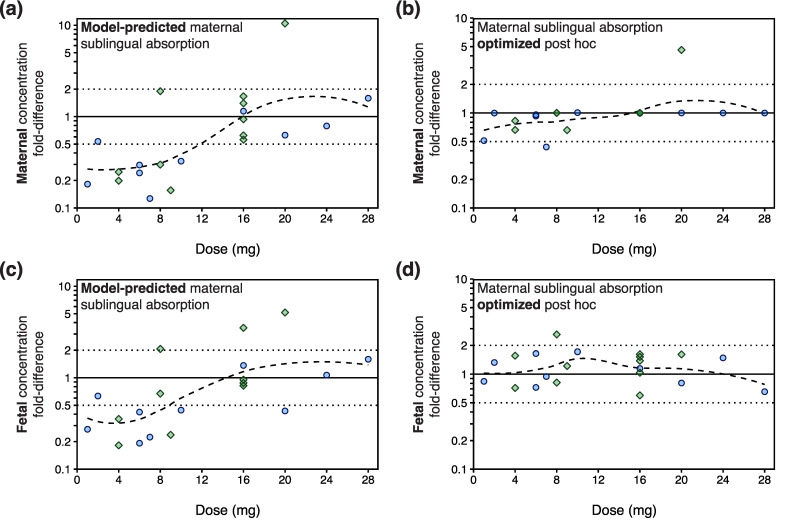
Goodness-of-fit plots for the maternal–fetal physiologically based pharmacokinetic (PBPK) model for buprenorphine, showing (**a**,**b**) dose vs. the ratio between maternal predicted and observed concentrations (concentration fold-difference) and (**c**,**d**) dose vs. fetal concentration fold-differences. Concentrations were predicted either (**a**,**c**) under the presumption that the proportion of the administered dose sublingually absorbed by the expectant mother equals 0.754 × (38.1 − 19.7 × *log*(Dose)), which is the default absorption extent in the maternal–fetal PBPK model for sublingual tablets, or (**b**,**d**) with the degree of maternal sublingual absorption optimized (across a range of 0.1–99.9%) post hoc to capture the reported maternal concentration–time point as accurately as possible. Blue circles (●) and green diamonds (◆) represent concentration fold-differences obtained from maternal and fetal concentration–time data reported by Bartu et al. [31] and Wiegand et al. [32], respectively. Dotted lines represent the 2-fold prediction error range. Curved dashed lines represent locally estimated scatterplot smoothing (LOESS) curves.

Despite that the nonlinear absorption model for sublingual tablets incorporated higher transmucosal absorption for lower doses, the general underprediction of maternal concentrations seemed to be driven by the underprediction of maternal exposure following administration of doses below 10 mg (Figure 6a). This reverberated to fetal concentration predictions, which were similarly underpredicted following maternal administration of doses below 10 mg (Figure 6c). The aforementioned post hoc optimization of maternal sublingual absorption resulted in an unbiased prediction of umbilical cord blood concentrations, where fetal concentration fold-differences were consistently close to unity across a 1–28 mg dose range (Figure 6d).

## 4. Discussion

In this study, a maternal–fetal PBPK model for sublingual buprenorphine was successfully developed. To the best of our knowledge, this is the first maternal–fetal PBPK for buprenorphine constructed to date. Pregnancy induces a plethora of physiological and metabolic changes that influence the maternal PK of buprenorphine [15]. Among others, the model incorporated reduced sublingual buprenorphine absorption driven by more acidic saliva during pregnancy and induced enzyme expression, thereby adequately predicting maternal PK during the second trimester, third trimester, and postpartum period. Maternal and fetal circulations were separated by a permeability-limited placenta model, where placental diffusion clearance was parameterized using buprenorphine’s physicochemical properties. When the degree of maternal sublingual absorption was individualized post hoc based on observed maternal concentrations to obtain a more realistic prediction in each pregnant woman, umbilical cord blood concentrations at delivery were adequately captured in the corresponding fetus. Modeling and simulation indicated that, as placenta volume and villous surface area increase, fetal buprenorphine exposure increases throughout gestation, despite progressively lower maternal concentrations (Figure 4).

On average, the maternal–fetal PBPK model, linked with a nonlinear absorption profile that described the degree of transmucosal absorption across sublingual tablet dose, adequately captured maternal PK during pregnancy following the administration of 8 mg tablets (Figure 2). However, PK variability was less well represented in the model, especially towards the end of the dosing interval. In addition, the model struggled with predicting single individual maternal concentration–time points across a larger dose range (Figure 3). Various factors can help explain this result. First, individual sublingual absorption of buprenorphine is highly variable. Bullingham et al. reported that the absolute bioavailability of 0.8 mg sublingual tablets in five subjects ranged between 15.7 and 94.4% [33]. Second, goodness-of-fit plots indicated that maternal concentrations following administration of especially lower-dosed sublingual tablets were poorly predicted, despite nonlinear absorption profiles assuming, by design, greater absorption from lower-dosed buprenorphine formulations. Lastly, the assessment of maternal concentration fold-differences vs. time between last maternal dose and blood sampling suggested that underprediction was more common between 12 and 24 h after dosing (Figure S4). A similar trend was observed in the data of Zhang et al. [29], where buprenorphine concentrations seemed to rise between 8 and 12 h (Figure 2). Individual buprenorphine concentrations fluctuate significantly during the terminal elimination phase, which is presumably caused by enterohepatic recirculation [34]. Enterohepatic recirculation was not assumed in any of the simulations, which may help explain the underprediction of buprenorphine concentrations towards the end of the dosing interval. Additionally, the contribution of enterohepatic recirculation could account for underpredicting maternal concentration–time points, despite assuming virtually complete (99.9%) sublingual absorption in some cases. Still, underprediction seemed to be driven particularly by an underestimation of the sublingual absorption degree from lower-dosed buprenorphine tablets, since there was no correlation found between dose and time between last maternal dose and blood sampling (*R*^2^ = 0.03, *p* = 0.273, Figure S4). Correcting post hoc for the likely extent of individual maternal sublingual absorption greatly improved the prediction of fetal buprenorphine concentrations. We therefore recommend prospectively determining maternal buprenorphine concentrations during pregnancy to allow adequate forecasting of fetal buprenorphine exposure using the developed maternal–fetal PBPK model.

The effect of decreased salivary pH on the sublingual absorption of buprenorphine was assessed by analyzing data reported by Mendelson et al. [23]. In this study, participants received a 2 mg buprenorphine solution. Linear regression indicated that at pH 7.01, which is the average pH in nonpregnant women [22], 45.5% of the 2 mg sublingual solution dose undergoes transmucosal absorption. A nearly identical proportion is estimated when employing our previously developed nonpregnancy nonlinear absorption model for buprenorphine sublingual solution (proportion equals 53.3 − 25.6 × *log*(Dose)) [19], namely 45.6%. This substantiates the validity of the linear regression analysis and nonpregnancy nonlinear absorption model.

In the current study, placental diffusion clearance was estimated using buprenorphine’s PSA and HBD properties. Simulated fetal exposure using the calculated clearance (at term CL_PD_ = 0.1166 L/h/mL placenta) seemed to be in line with umbilical cord blood samples obtained at delivery. Although a true fetal C_max_ was likely not measured, concentration–time points closest to the anticipated C_max_ in umbilical cord blood were adequately captured (Figure S3c), indicating that the estimated placental diffusion clearance was acceptable. Nanovskaya et al. determined placental clearance of buprenorphine using ex vivo placenta perfusion models. The results were either normalized to antipyrine [35], thereby complicating the manual calculation of the placental clearance of buprenorphine, or an unusually low placental clearance was reported (0.29 mL/min/cotyledon, which at term gestation corresponds to CL_PD_ = 0.00044 L/h/mL placenta, according to default Simcyp formulae) [36], which resulted in illogical simulated fetal concentration–time profiles. Hence, we opted to calculate placental diffusion clearance using buprenorphine’s physicochemical properties. It should be noted, however, that the formula to predict transplacental passive permeability (Equation (2)) was, in fact, developed to describe passive permeability through the intestinal membrane [27]. Nonetheless, applying the formula to calculate transplacental passive permeability is not uncommon [18]. Moreover, active transporters of buprenorphine have not been identified to date [37], and buprenorphine likely crosses the placenta through passive diffusion [36]; thus, we believe the assumption of passive permeability is justified.

Simcyp, by default, incorporates pregnancy-associated induction profiles for the expression of hepatic CYP3A4, hepatic UGT1A1, and intestinal UGT1A1 [26]. In addition, in the present study, the increased expression of hepatic CYP2C8, intestinal CYP3A4, and hepatic UGT2B7 during pregnancy was assumed. Whether CYP2C8-mediated clearance increases clinically during pregnancy remains to be determined. However, an in vitro study demonstrated the pregnancy-related hormone progesterone induces CYP2C8 to a similar degree as CYP3A4 in human hepatocytes [38]. Hence, the same pregnancy-associated induction profiles for hepatic CYP2C8 and CYP3A4 were assumed. Likewise, clearance of UGT2B7 substrates morphine and zidovudine increases by approximately 1.5-fold during pregnancy [15,39,40], which indicates the induction of hepatic UGT2B7 to a similar degree as assumed within the Simulator for UGT1A1. The default pregnancy-associated induction profile of hepatic UGT1A1 was therefore copied to UGT2B7. It should be noted, however, that both morphine and zidovudine are intermediate-to-high hepatic extraction drugs (ratios of 0.7 and 0.62, respectively) [41,42]. The observed increase in clearances could therefore be, in part, explained by greater hepatic perfusion during pregnancy [43]. It is unclear whether the expression of intestinal CYP3A4 increases throughout gestation in humans. Although mouse data indicate that intestinal CYP3A4 expression is not affected by pregnancy [44], we nonetheless assumed the same pregnancy-associated induction profile for intestinal CYP3A4 as for its hepatic counterpart, since it is difficult to clinically discern the site (hepatic, intestinal, or both) responsible for increased CYP3A4-mediated metabolism. Given this uncertainty, we performed a sensitivity analysis to investigate the effect of buprenorphine metabolizing enzyme abundances on maternal apparent clearance (Figure S5). The results indicated that the impact of intestinal CYP3A4 expression is insignificant (7.5% increase in apparent clearance under 5-fold the usual expression).

Certain limitations apply to the developed maternal–fetal PBPK model. First, as with many maternal–fetal PBPK models, the prediction of fetal PK has only been assessed at delivery. Although the model scales maternal–fetal physiology and transplacental exchange throughout pregnancy, fetal predictions at any point before delivery could not be verified due to lack of clinical data. Second, the effect of decreased salivary pH during pregnancy on the transmucosal absorption of buprenorphine was assessed using data obtained from subjects who received a 2 mg sublingual solution. The nonlinear absorption profiles of the maternal–fetal PBPK model assume the absorption effect of lower salivary pH is the same for both formulations (sublingual tablets and solution) and all doses. Third, a Kp scalar was applied to describe the maternal and fetal tissue distribution. The Kp scalar value was empirically established to recover the Vss of the previously developed nonpregnant adult PBPK model [19] and lacks an experimental foundation and validation. Fourth, the existing maternal–fetal PBPK model lacks the capability to characterize the pharmacokinetics of buprenorphine metabolites, including norbuprenorphine, which are anticipated to play a role in the severity of NOWS among exposed fetuses [45]. Finally, the current fetal module of Simcyp does not allow simulation outside the 15–40 weeks’ gestational age range, and we additionally assumed that postpartum women were essentially the same as nonpregnant women. A more gradual return to pre-pregnancy physiology during puerperium seems more plausible, but this could not be modeled due to software limitations. Regarding the postpartum sublingual absorption of buprenorphine, salivary pH increases to pre-pregnancy values quickly following delivery [46]. We therefore believe the correction factor of 0.754 for sublingual absorption is unwarranted for simulation during the postpartum period.

## 5. Conclusions

The adequate forecasting of fetal buprenorphine concentrations throughout gestation may elucidate the effect of prenatal buprenorphine exposure on NOWS severity. The maternal–fetal PBPK model developed in this study forms the springboard for this endeavor. By measuring maternal buprenorphine plasma concentrations during pregnancy, and subsequent tailoring of the maternal PBPK model prediction, an adequate impression of fetal exposure can be obtained. A future clinical study could employ modeling and simulation to forecast fetal buprenorphine exposure in opioid-dependent pregnant women maintained on buprenorphine at different moments during gestation and investigate the correlation with NOWS severity. This, in turn, can help refine the treatment of NOWS by tailoring treatment to the expected severity of NOWS, thereby improving the clinical care of vulnerable newborns affected by the opioid crisis.

## Data Availability

The data presented in this study are available upon written and reasonable request from the corresponding authors.

## Supplementary Materials

The following supporting information can be downloaded at https://www.mdpi.com/article/10.3390/pharmaceutics16030375/s1, Figure S1: Fold-differences in buprenorphine Kp for the organs and tissues included in the maternal section of the maternal–fetal PBPK model as a function of gestational age; Figure S2: Recovery of buprenorphine in saliva across salivary pH; Figure S3: Maternal–fetal physiologically based pharmacokinetic (PBPK) model-based predicted and observed maternal and fetal buprenorphine plasma concentrations at delivery in mother–fetus dyads grouped together if expectant mothers received the same buprenorphine dosing regimen; Figure S4: Goodness-of-fit plots for the maternal–fetal PBPK model for buprenorphine; Figure S5: Sensitivity of the maternal–fetal PBPK model-based predicted maternal CL/F to changes in enzyme abundance; Table S1: Input data for the full maternal–fetal physiologically based pharmacokinetic (PBPK) model for buprenorphine; Table S2: Predicted and observed buprenorphine pharmacokinetic parameters following administration of sublingual tablets (8 mg twice daily) during pregnancy. References [19,21,23,24,25,28,29,30,31,32,47,48,49,50,51,52,53,54,55,56] are cited in the Supplementary Materials.
