# Supplementary material for: Forecasting Fetal Buprenorphine Exposure through Maternal–Fetal Physiologically Based Pharmacokinetic Modeling
Source: Pharmaceutics. 2024 Mar 8;16(3):375. doi: 10.3390/pharmaceutics16030375 (PMC10975492; doi:10.3390/pharmaceutics16030375)
Supplement: Supplementary file 1 [file pharmaceutics-16-00375-s001.zip › pharmaceutics-2893439-supplementary.pdf]

Supplementary Materials

Forecasting Fetal Buprenorphine Exposure through Maternal–Fetal Physiologically Based Pharmacokinetic Modeling

Matthijs W. van Hoogdalem [1,2,†], Ryota Tanaka [1,†], Khaled Abduljalil [3], Trevor N. Johnson [3], Scott L. Wexelblatt [4,5,6], Henry T. Akinbi [4,5], Alexander A. Vinks [1,5,6] and Tomoyuki Mizuno [1,5,6,*]

[1] Division of Translational and Clinical Pharmacology, Cincinnati Children's Hospital Medical Center, Cincinnati, OH 45229, USA
[2] James L. Winkle College of Pharmacy, University of Cincinnati, Cincinnati, OH 45229, USA
[3] Certara UK Limited, Simcyp Division, Sheffield S1 2BJ, UK
[4] Division of Neonatology, Perinatal Institute, Cincinnati Children's Hospital Medical Center, Cincinnati, OH 45229, USA
[5] Department of Pediatrics, College of Medicine, University of Cincinnati, Cincinnati, OH 45267, USA
[6] Center for Addiction Research, College of Medicine, University of Cincinnati, Cincinnati, OH 45267, USA
* Correspondence: tomoyuki.mizuno@cchmc.org; Tel.: +1-(513)-636-0912
† These authors contributed equally to this work.

Table S1. Input data for the full maternal-fetal physiologically-based pharmacokinetic (PBPK) model for buprenorphine.

Parameter	Value
Physicochemical	
Molecular weight (g/mol)	467.6 [1]
Log*P*	4.98 [2]
Compound type	Ampholyte [2]
pK_a (acid; phenol)	9.62 [2]
pK_a (base; amine)	8.31 [2]
Blood binding	
Blood-to-plasma ratio	1 [3]
$f_{u, plasma}$	0.04 [4]
Plasma binding components	AGP [5]
Gastrointestinal tract absorption (first order model)	
f_a	1[a]
k_a (h^{-1})	0.016 [6]
Lag time (h)	0.22 [6]
$f_{u, gut}$	0.4 [6]
Q_{gut} (L/h)	16.8[b]
$P_{eff, man}$ (10^{-4} cm/s)	6.83[b]
Caco-2 7.4:7.4 (10^{-6} cm/s)	66.7 [7]
Lung[c] absorption (first order model)	
f_a	1[a]
k_a (h^{-1})	1 [6]
Proportion of dose inhaled[c]$_{sublingual\ tablet}$ (%)	0.754 × (38.1 – 19.7 × *log*(Dose))[d]
Proportion of dose inhaled[c]$_{sublingual\ solution}$ (%)	0.754 × (53.3 – 25.6 × *log*(Dose))[d]
Maternal distribution (full PBPK model)	
Tissue-to-plasma partition coefficients (Kp)	
Adipose[e]	17.800 [5]
Bone[f]	1.603 [5]
Brain[f]	19.206 [5]
Gut[g]	2.252 [5]
Heart[f]	1.714 [5]
Kidney[g]	6.372 [5]
Liver	4.742[h]
Lung[f]	3.921 [5]
Muscle[f]	0.905 [5]
Pancreas[f]	3.016 [5]
Skin	3.500 [8]
Spleen[f]	2.286 [5]
Feto-placenta	2.914[h]
Fetal distribution (full PBPK model)	
Kp values calculated as described by Rodgers and Rowland (method 2) [9,10].	
Kp scalar	0.26819[i]
Elimination	
CYP2C8	
V_{max} (pmol/min per mg protein)	176.3 [11]
K_m (μM)	12.4 [11]
CYP3A4	
V_{max} (pmol/min per mg protein)	520 [11]
K_m (μM)	13.6 [11]
UGT1A1	
V_{max} (pmol/min per mg protein)	2870 [12]
K_m (μM)	66.4 [12]
UGT1A3	
V_{max} (pmol/min per mg protein)	286 [12]
K_m (μM)	202 [12]
UGT2B7	
V_{max} (pmol/min per mg protein)	173 [12]
K_m (μM)	13.8 [12]
UGT2B17	
V_{max} (pmol/min per mg protein)	172 [12]
K_m (μM)	9.6 [12]
$f_{u, mic}$	0.1 [13]
CL_{renal} (L/h)	0.54[j]
$CL_{biliary}$ (μl/min per million hepatocytes)	51 [14]
Transport	
Placenta	
CL_{PDM} (L/h/mL placenta)	0.1166[k]
CL_{PDF} (L/h/mL placenta)	0.1166[k]

AGP, α1-acid glycoprotein; $CL_{biliary}$, biliary clearance; CL_{PDM}, clearance between maternal blood and placenta cell; CL_{PDF}, clearance between placenta cell and fetal blood; CL_{renal}, renal clearance; CYP, cytochrome P450; f_a, fraction absorbed; $f_{u, gut}$, fraction unbound in enterocytes; $f_{u, mic}$, fraction unbound in *in vitro* microsomal incubation; $f_{u, plasma}$, fraction unbound in blood plasma; k_a, first-order absorption rate constant; K_m, Michaelis-Menten constant; $P_{eff, man}$, human jejunum effective permeability; Q_{gut}, nominal flow in gut model; UGT, UDP-glucuronosyltransferase; V_{max}, maximum metabolic rate.

[a]Assumed value. [b]Simcyp predicted value. [c]The sublingual route of administration is not available in Simcyp; sublingual absorption is therefore mimicked by employing the first-order inhalation model in combination with the inhaled route of administration. [d]Formula described by van Hoogdalem *et al.* [6], multiplied by 0.754 to correct for lower salivary pH during pregnancy; more details are provided in this publication. Dose is in mg and logarithm base is 10. The value is calculated manually and the computed proportion is then entered into the first-order inhalation model. Note that a coefficient of variation (CV) of 33.9% is applied to the administered dose to reflect variability in bioavailability. [e,f,g]Reported radioactivity at 24, 8, and 1 h post-injection was used for calculation, respectively. Note that these are base values that need to be multiplied by gestational age-dependent Kp fold-differences reported in the main text. [h]Calculated as described by Rodgers and Rowland (method 2 in Simcyp) [9,10], with a Kp scalar of 0.26819 applied. [i]Kp scalar of 0.26819 was empirically obtained by assessing which Kp scalar is needed to recover volume of distribution at steady-state (V_{ss}) of 6.23 L/h, the base model's default value [6], when all maternal Kp values are calculated following method 2. [j]Calculated by Johnson *et al.* [14] based on a mass balance study where 1% was excreted unchanged in urine [15], with total plasma clearance of 54.1 L/h [3]. [k]Placenta diffusion clearances at term (40 weeks gestational age); estimated in Simcyp using buprenorphine's hydrogen bond donor (HBD; count = 2) [1] and polar surface area (62.2 Å2) [1]

Information (see for more details main text).

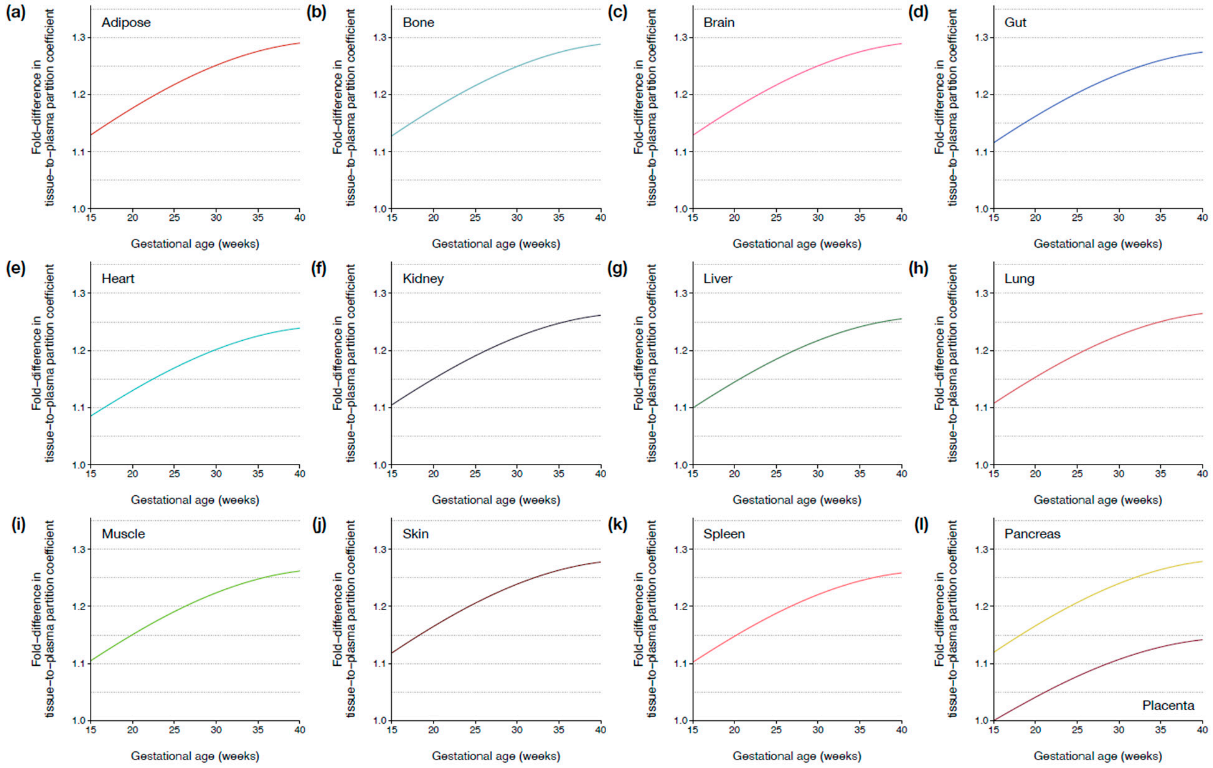

Figure S1. Fold-differences in buprenorphine tissue-to-plasma partition coefficients (Kp) for the organs and tissues included in the maternal section of the maternal-fetal physiologically-based pharmacokinetic (PBPK) model as a function of gestational age. Kp values were calculated as described by Rodgers and Rowland (method 2 in Simcyp) [9,10].

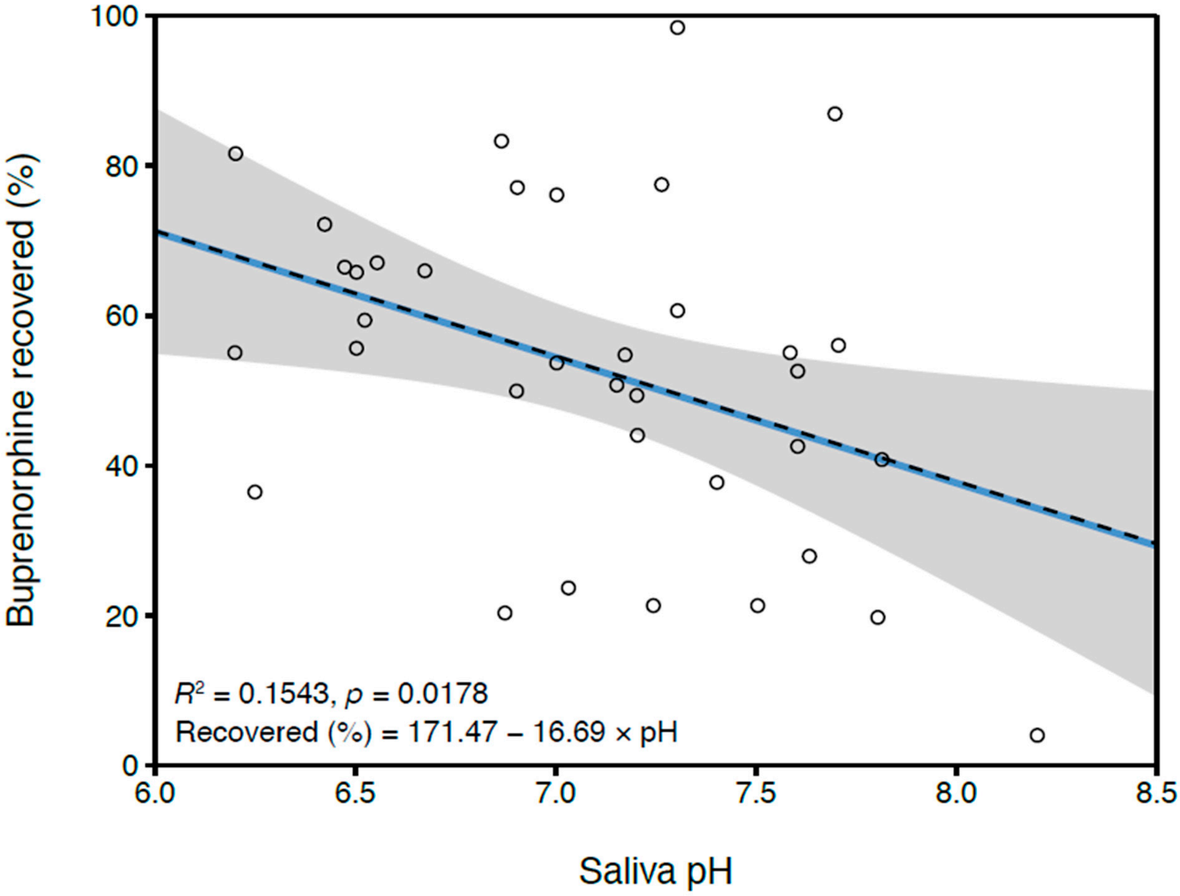

Figure S2. Recovery of buprenorphine in saliva across salivary pH. Open circles represent data reported by Mendelson *et al* [16]. Dashed line with gray shaded area represent linear regression with 95% confidence interval, respectively, where the coefficient of determination (R^2), p value, and linear regression model are denoted in the lower left corner. Blue solid line represents the linear regression line originally reported by Mendelson *et al* [16].

Table S2. Predicted and observed buprenorphine pharmacokinetic parameters following administration of sublingual tablets (8 mg twice daily) during pregnancy.

Clinical trial	Gestational age	*n*	Mean age (years)		AUC_{0-12h} (ng×h/mL)	CL/F (L/h)	C_{max} (ng/mL)	T_{max} (h)
Zhang *et al.* [17,18]	22 weeks (second trimester)	4	27.3	Predicted	17.6	622.7	3.35	0.73
				Observed	15.4	520.0	3.80	1.00
				**P/O ratio**	1.14	1.20	0.88	0.73
Zhang *et al.* [17,18]	33.9 weeks (third trimester)	4	27.9	Predicted	15.5	701.5	3.08	0.74
				Observed	12.4	644.8	2.62	0.47
				**P/O ratio**	1.25	1.09	1.17	1.58
Zhang *et al.* [17,18]	Postpartum (7.4 weeks following childbirth)	10	28.6	Predicted	26.4	357.3	5.14	0.99
				Observed	27.9	286.4	5.40	0.91
				**P/O ratio**	0.95	1.25	0.95	1.09

AUC_{0-12h}, area under the curve from 0 to 12 hours; CL/F, apparent clearance; C_{max}, peak concentration; P/O ratio, ratio between predicted and observed value; T_{max}, time to reach C_{max}.

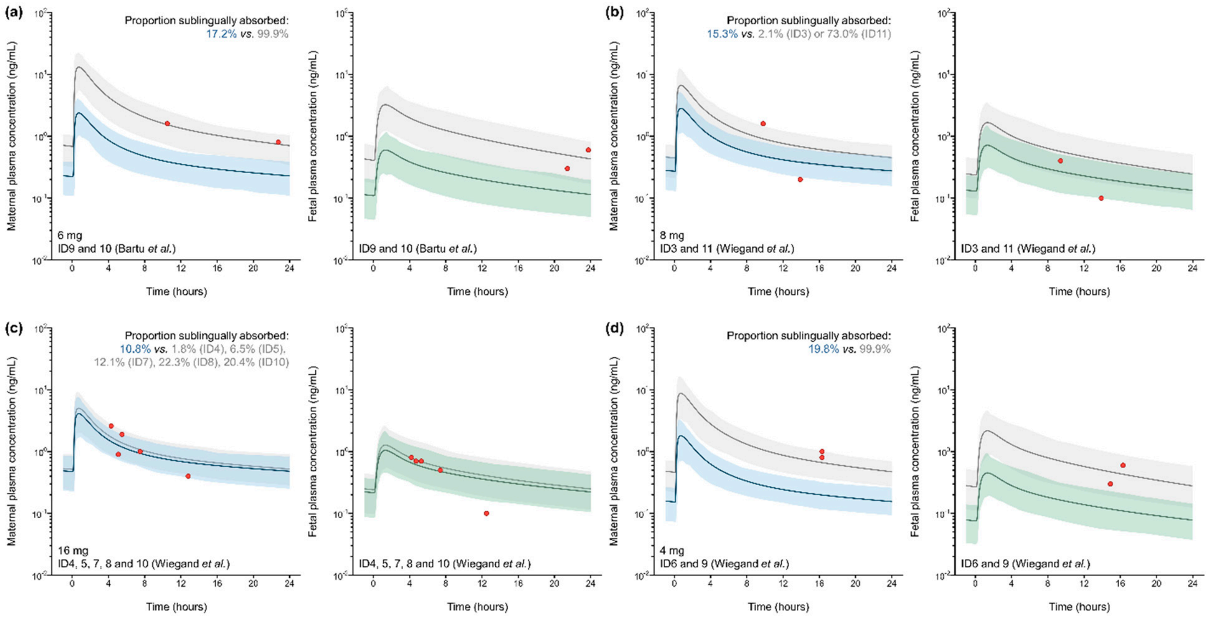

Figure S3. Maternal-fetal physiologically-based pharmacokinetic (PBPK) model-based predicted and observed maternal and fetal buprenorphine plasma concentrations at delivery in mother-fetus dyads grouped together if mothers received the same buprenorphine dosing regimen. Individual doses are shown in the lower left corner of each figure subsection. Closed red circles represent observed buprenorphine concentrations in maternal and umbilical cord blood reported by (**a**) Bartu *et al*. [19] and (**b–d**) Wiegand *et al*. [20]. Simulated concentration-time profiles with 5th to 95th population percentile ranges (n = 100 mother-fetus dyads) were created either under the presumption that the proportion of the administered dose sublingually absorbed by the expectant mother equals 0.754 × (38.1 – 19.7 × *log*(Dose)), which is the default absorption extent in the maternal-fetal PBPK model for sublingual tablets (shown in blue and green for maternal and fetal concentrations, respectively), or with the degree of maternal sublingual absorption optimized (across a range of 0.1–99.9%) *post hoc* to capture the reported maternal concentration-time point as accurately as possible, followed by averaging the individual profiles of the same dosing regimen (shown in grayscale).

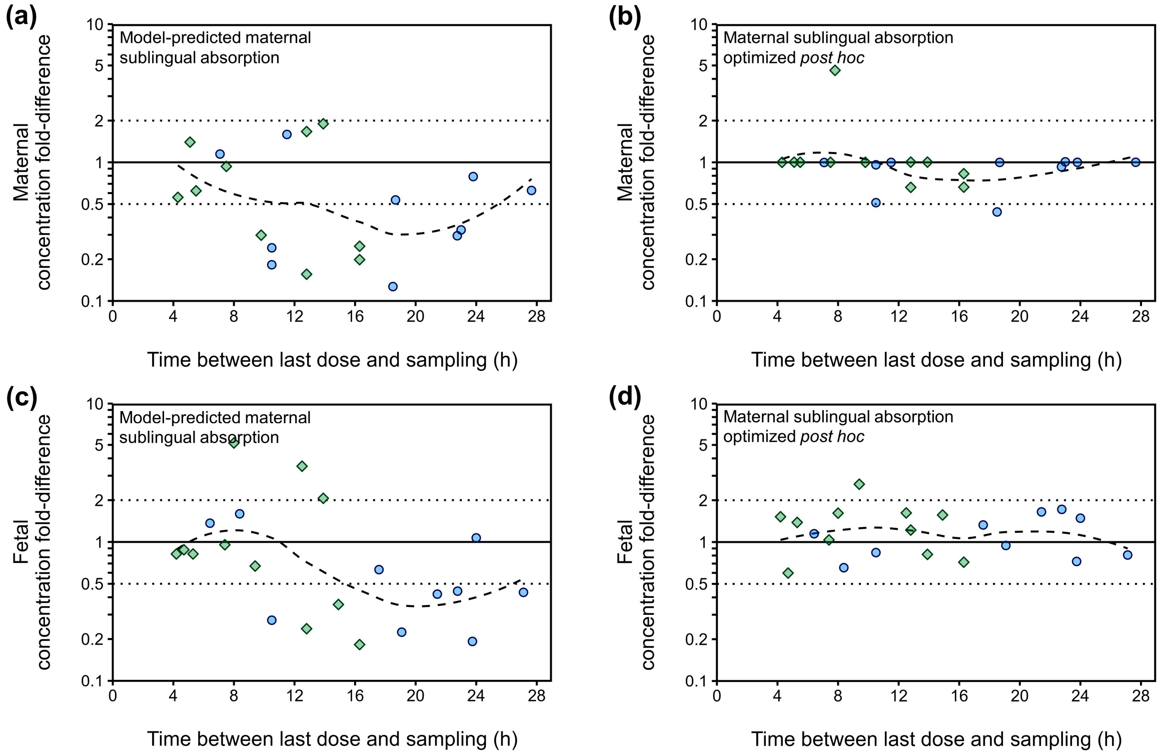

Figure S4. Goodness-of-fit plots for the maternal-fetal physiologically-based pharmacokinetic (PBPK) model for buprenorphine, showing **(a,b)** time between last dose and sampling *vs*. the ratio between maternal predicted and observed concentrations (concentration folddifference) and **(c,d)** time between last dose and sampling *vs*. fetal concentration fold-differences. Concentrations were predicted either **(a,c)** under the presumption that the proportion of the administered dose sublingually absorbed by the mother equals 0.754 × (38.1 – 19.7 × *log*(Dose)), which is the default absorption extent in the maternal-fetal PBPK model for sublingual tablets, or **(b,d)** with the degree of maternal sublingual absorption optimized (across a range of 0.1–99.9%) *post hoc* to capture the reported maternal concentration-time point as accurately as possible. Blue circles (●) and green diamonds (◆) represent concentration fold-differences obtained from maternal and fetal concentration-time data reported by Bartu *et al*. [19] and Wiegand *et al*. [20], respectively. Dotted lines represent the 2-fold prediction error range. Curved dashed lines represent locally estimated scatterplot smoothing (LOESS) curves.

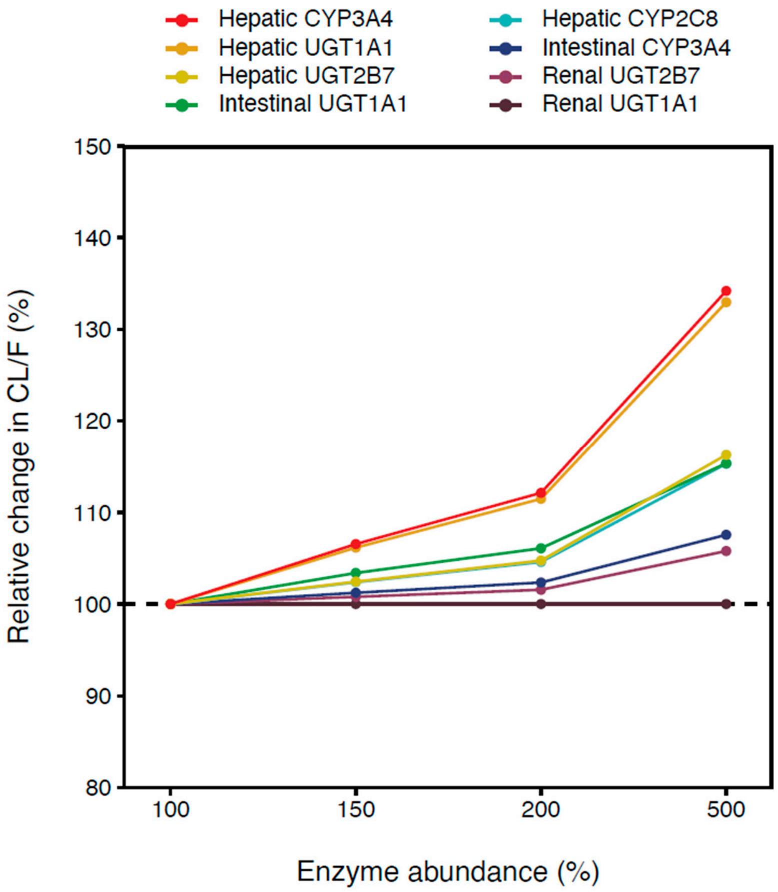

Figure S5. Sensitivity of the maternal-fetal physiologically-based pharmacokinetic (PBPK) model-based predicted maternal apparent clearance (CL/F) to changes in enzyme abundance. All maternal pregnancy-associated enzyme induction profiles were disabled in Simcyp, and enzyme abundance was manually changed in the population file to investigate the effect on CL/F. The effect of enzyme abundance on maternal CL/F is a marker of the effect pregnancy-associated enzyme induction profiles have.

Reference

1. NCBI. National Center for Biotechnology Information: PubChem Compound Summary for CID 644073, Buprenorphine. 2022. <https://pubchem.ncbi.nlm.nih.gov/compound/644073> Accessed 20 April 2022.
2. Avdeef, A.; Barrett, D.A.; Shaw, P.N.; Knaggs, R.D.; Davis, S.S. Octanol-, chloroform-, and propylene glycol dipelargonat-water partitioning of morphine-6-glucuronide and other related opiates. *J Med Chem* **1996**, *39*, 4377-4381.
3. Bullingham, R.E.; McQuay, H.J.; Moore, A.; Bennett, M.R. Buprenorphine kinetics. *Clin Pharmacol Ther* **1980**, *28*, 667-672.
4. Elkader, A.; Sproule, B. Buprenorphine: clinical pharmacokinetics in the treatment of opioid dependence. *Clin Pharmacokinet* **2005**, *44*, 661-680.
5. Takahashi, Y., Ishii, S., Arizono, H., Nishimura S, Tsuruda, K., Saito N., Nemoto, H., Jin, Y., Esumi, Y. [Pharmacokinetics of buprenorphine hydrochloride (BN•HCl) (1): absorption, distribution, metabolism and excretion after percutaneous (TSN-09: BN•HCl containing tape application) or subcutaneous administration of BN•HCl in rats]. *Drug Metab. Pharmacokinet.* **2001**, *16*, 569-583.
6. van Hoogdalem, M.W., Johnson, T.N., Vinks, A.A. & Mizuno, T. Development and validation of a full physiologically-based pharmacokinetic model for sublingual buprenorphine that accounts for nonlinear bioavailability. [Preprint]. *Authorea.* **2022**.
7. Hassan, H.E.; Myers, A.L.; Coop, A.; Eddington, N.D. Differential involvement of P-glycoprotein (ABCB1) in permeability, tissue distribution, and antinociceptive activity of methadone, buprenorphine, and diprenorphine: in vitro and in vivo evaluation. *J Pharm Sci* **2009**, *98*, 4928-4940.
8. Holland, M.J.; Carr, K.D.; Simon, E.J. Pharmacokinetics of [3H]-buprenorphine in the rat. *Res Commun Chem Pathol Pharmacol* **1989**, *64*, 3-16.

9. Rodgers, T.; Rowland, M. Physiologically based pharmacokinetic modelling 2: predicting the tissue distribution of acids, very weak bases, neutrals and zwitterions. *J Pharm Sci* **2006**, *95*, 1238-1257.
10. Rodgers, T.; Rowland, M. Mechanistic approaches to volume of distribution predictions: understanding the processes. *Pharm Res* **2007**, *24*, 918-933.
11. Picard, N.; Cresteil, T.; Djebli, N.; Marquet, P. In vitro metabolism study of buprenorphine: evidence for new metabolic pathways. *Drug Metab Dispos* **2005**, *33*, 689-695.
12. Chang, Y.; Moody, D.E. Glucuronidation of buprenorphine and norbuprenorphine by human liver microsomes and UDP-glucuronosyltransferases. *Drug Metab Lett* **2009**, *3*, 101-107.
13. Cubitt, H.E.; Houston, J.B.; Galetin, A. Relative importance of intestinal and hepatic glucuronidation-impact on the prediction of drug clearance. *Pharm Res* **2009**, *26*, 1073-1083.
14. Johnson, T.N.; Jamei, M.; Rowland-Yeo, K. How Does In Vivo Biliary Elimination of Drugs Change with Age? Evidence from In Vitro and Clinical Data Using a Systems Pharmacology Approach. *Drug Metab Dispos* **2016**, *44*, 1090-1098.
15. Pharmaceuticals., R.C. NDA: 20-733 Suboxone® sublingual tablets – Clinical pharmacology/biopharmaceutics review. Richmond, VA: Reckitt & Colman. **2000**.
16. Mendelson, J.; Upton, R.A.; Everhart, E.T.; Jacob, P., 3rd; Jones, R.T. Bioavailability of sublingual buprenorphine. *J Clin Pharmacol* **1997**, *37*, 31-37.
17. Zhang, H.; Kalluri, H.V.; Bastian, J.R.; Chen, H.; Alshabi, A.; Caritis, S.N.; Venkataramanan, R. Gestational changes in buprenorphine exposure: A physiologically-based pharmacokinetic analysis. *Br J Clin Pharmacol* **2018**, *84*, 2075-2087.
18. Bastian, J.R.; Chen, H.; Zhang, H.; Rothenberger, S.; Tarter, R.; English, D.; Venkataramanan, R.; Caritis, S.N. Dose-adjusted plasma concentrations of sublingual buprenorphine are lower during than after pregnancy. *Am J Obstet Gynecol* **2017**, *216*, 64.e1-64.e7.

19. Bartu, A.E.; Ilett, K.F.; Hackett, L.P.; Doherty, D.A.; Hamilton, D. Buprenorphine exposure in infants of opioid-dependent mothers at birth. *Aust N Z J Obstet Gynaecol* **2012**, *52*, 342-347.
20. Wiegand, S.L.; Swortwood, M.J.; Huestis, M.A.; Thorp, J.; Jones, H.E.; Vora, N.L. Naloxone and Metabolites Quantification in Cord Blood of Prenatally Exposed Newborns and Correlations with Maternal Concentrations. *AJP Rep* **2016**, *6*, e385-e390.
